# Habitats and movement patterns of white whales *Delphinapterus leucas* in Svalbard, Norway in a changing climate

**DOI:** 10.1186/s40462-018-0139-z

**Published:** 2018-10-24

**Authors:** Jade Vacquié-Garcia, Christian Lydersen, Rolf A. Ims, Kit M. Kovacs

**Affiliations:** 1 Norwegian Polar Institute, Fram Centre, N-9296 Tromsø, Norway; 2 University of Tromsø, the Arctic University of Norway, Tromsø, Norway

**Keywords:** Adaptation, Arctic, Atlantification, Beluga, Climate change, Environmental change, Prey shifting, Space use

## Abstract

**Background:**

The Arctic is experiencing rapid reductions in sea ice and in some areas tidal glaciers are melting and retracting onto land. These changes are occurring at extremely rapid rates in the Northeast Atlantic Arctic. The aim of this study was to investigate the impacts of these environmental changes on space use by white whales (*Delphinapterus leucas*) in Svalbard, Norway. Using a unique biotelemetry data set involving 34 animals, spanning two decades, habitat use and movement patterns were compared before (1995–2001) and after (2013–2016) a dramatic change in the regional sea ice regime that began in 2006.

**Results:**

White whales were extremely coastal in both study periods, remaining near the islands within the Svalbard Archipelago, even when winter sea ice formation pushed them offshore somewhat (later in the year in the recent period), into areas with drifting sea ice (concentrations up to 90%). In both periods, the whales followed the same basic patterns seasonally; they occupied the west coast in summer and shifted to the east coast as winter approached. However, space use did change between the two periods, with the whales spending less time close to tidal glacier fronts in the second period compared to the first (2^nd^-36% vs 1^st^-51%), a habitat characterized by low swimming speeds and high turning angles, and more time out in the fjords (2^nd^-26% vs1^st^-10%). Use of coastal transit corridors remained the same in both periods; the whales appear to minimize time spent moving between fjords.

**Conclusions:**

Glacier fronts have previously been shown to be important foraging areas for white whales in Svalbard and the movement metrics documented in this study confirms that this is still the case. However, use of the Fjords habitat in summer and fall (frequency of occupancy and movement metrics) seen in the recent period suggests that the white whales might now also be feeding on Atlantic prey that is increasingly common in the fjords, concomitant with influxes of Atlantic Water along the west coast of Svalbard. Such behavioural flexibility, if confirmed by further diet studies, would likely be important for white whales in adapting to new conditions in Svalbard.

**Electronic supplementary material:**

The online version of this article (10.1186/s40462-018-0139-z) contains supplementary material, which is available to authorized users.

## Background

The Arctic is currently undergoing rapid environmental change, with sea ice losses and retraction of tidal glaciers being among the most visible changes to date [1, 2]. For endemic Arctic marine mammals, sea ice habitats have been low-competition environments that are sheltered from open-water predators and many potential human impacts as well as from inclement weather, which is particularly important for young animals [3, 4]. In addition, sea ice environments have provided a seasonally rich food supply, particularly in the marginal ice zone and at fast-ice edges, in predictable polynya areas [3, 4]. Similarly, tidal glacier fronts also have provided rich foraging grounds for these animals [2]. The reduction of sea ice cover and retraction of tidal glaciers are thus decreasing the available habitat for ice-associated marine mammal species and likely concomitantly affecting their behaviour. In recent years, our capacity to demonstrate impacts on ice-associated species is increasing, however it is still limited due to a general lack of long-term data series from most populations [5].

The Norwegian Arctic archipelago of Svalbard has experienced anomalously rapid increases in both air and sea water temperatures during the past two decades [6–8] and this area has had the greatest decrease in the seasonal duration of sea-ice cover within the circumpolar Arctic [5]. In 2006, the sea-ice regime in the Svalbard area underwent an unexpected collapse with dramatic changes in sea-ice conditions persisting to the present day [9]. Land-fast sea ice extent has declined sharply especially in fjords on the west coast of Svalbard. This is partly due to intrusion of water from the West Spitsbergen Current (WSC) that penetrates into fjords on the west coast of Svalbard more frequently; the WSC is also warming [9, 10]. Concomitantly, tidal glaciers in Svalbard are retracting [2]. The current overall mass balance for Svalbard glaciers is negative [11–16] and Svalbard glaciers are expected to continue to melt and retreat in the future. In combination, these changes in both sea ice and glacier front areas has led to quite negative projections regarding future impacts on ice-associated marine mammals in the region (e.g. [17]) and also make Svalbard a particularly interesting area to study.

White whales, *Delphinapterus leucas,* are an ice-associated marine mammal that has a circumpolar Arctic distribution. Globally, there are thought to be some 150,000 individuals, occurring in 20 recognized ‘stocks’. The species is listed on the IUCN (International Union for Conservation of Nature) Red List as least concern, except the Cook Inlet subpopulation, which is listed as critically endangered [18]. Since the early 1990s, distribution and movement patterns of the species have been collected from various parts of the Arctic using satellite telemetry [19–27]. Highly variable movement patterns have been found for the various stocks, with some stocks undertaking seasonal migrations to varying extents, with distances travelled ranging from tens of kilometres [22] to several hundreds of kilometres [24], while others stocks reside in the same locality all year round [28, 29]. In the Svalbard Archipelago, white whales are year-round residents [25] and this species is the most frequently observed Arctic cetacean in the area [30]. Satellite tracking in this region in the late 1990s showed that during ice-free periods of the year, white whales spent more than 50% of their time in front of tidal glaciers and when they moved between glacier fronts they did so in an extreme coastal, directed manner [25]. Fatty acid analyses of the blubber of white whales in Svalbard suggests that polar cod, *Boreogadus saida,* and capelin*, Mallotus villosus,* two species particularly common in the cold high-productivity glacial fronts areas, were the main components of their diet in the late 1990s [31]. White whales’ close affiliation to glaciers fronts, as well as potential changes in their diet, warrant investigation given that their primary foraging habitat has been diminishing rapidly due to glacial melting and to intrusion of warmer water during the last decade. Thus, the aim of the present study was to use data from satellite tracking to compare habitat use and movement patterns of white whales in Svalbard before and after the major changes in sea ice conditions that commenced in 2006.

## Methods

### Data logger deployments and data collection

Field work was conducted in several fjords on Spitsbergen, the largest island within the Svalbard Archipelago, Norway, during the summers of 1995–2001 and 2013–2016. White whales were live-captured using a nylon net (100 × 8 m, mesh size 50 cm) set from beaches [25]. Sex and age were determined based on examination of the genitalia, body size and skin colour [32] and confirmed genetically based on DNA from skin samples in the DNA-laboratory at Bioforsk Svanhovd (Svanvik, Norway). Among the 76 captured individuals, only adult animals (white skin colour) were instrumented with either Satellite Relay Data Loggers (SRDLs) or Conductivity-Temperature-Depth Satellite Relay Data Loggers (CTD-SRDLs) (Sea Mammal Research Unit, University of St Andrews). Both types of loggers collect and transmit information on location via the Argos satellite system (for details; see [33]). Locations are estimated by the orbiting satellites and a location class (LC; Z/B/A/1/2/3), associated with an error, is assigned to each position [33]. Data were sent whenever a tag made contact with a satellite; no duty cycling was enacted.

In the first study period (1995–2001), the data loggers had 2 flexible straps made of PVC impregnated belt material cast into their undersides and the satellite tags were attached by placing the flexible belt material transversely on each side of the whale’s dorsal ridge (see [25] for more details). In the latter study period (2013–2016), the belting material was replaced with thin plastic covered wires to reduce drag and the data loggers were smaller, lighter and more streamlined (Additional file 1: Figure S1). These small improvements in tag design increased the longevity of data records, but it is unlikely that either tag type had significant impacts on the whales given the small tag:body mass ratio. A total of 38 individuals, 34 males and four females were instrumented during these two periods. Due to the low number of females, only data for males were analysed herein (18 males from the period 1995–2001 and 16 males from the period 2013–2016).

### Data processing

#### Track filtration

All data processing and modelling was done using the R statistical framework (R Development Core Team 2010). Satellite-derived locations were first filtered using a speed, distance and angle filter (SDA filter; [34]) using the R package “argosfilter” [35]. This filter removes all LC Z values and locations requiring unrealistic swimming speeds or unlikely turning angles [34]. The swimming speed threshold was set at 3 m/s and all spikes with angles smaller than 15 or 25 degrees were removed if their lengths were greater than 2.5 or 5 km, respectively [23]. Because white whales in the Svalbard area are extremely coastal (see [25]), locations were further processed using a simplified particle filter correcting for “on-land” positions (see more details on the standard particle filter in [36]). Each of the filtered locations was first classified as an at-sea or on-land location using a land mask. In order to take into account the dynamics of the Svalbard coastline, which includes glacier fronts that can undergo retractions as well as rapid surges, two different land/glaciers shapefiles were used for masking of the 1995–2001 tracks and of the 2013–2016 tracks, respectively (Norwegian Polar Institute (NPI), www.npolar.no and [37]). Fifty particles were then created for each on-land location, within a radius based on each location’s Argos error. Argos errors used for the various LCs were taken from [38] for animals tagged in 1995–2001 and from [39] for animals tagged in 2013–2016. Each of the created particles was then classified as an at-sea or on-land particle. Finally, each initial on-land location was corrected based on the geographic average of all of its associated at-sea particles. In cases where an initial on-land location still remained on land after the correction, it was removed from the analyses.

Because location data were sampled independently of the speed along the track, filtered and corrected locations were subsequently interpolated such that they were regularly spaced at 1 h intervals along the track-line. If there was more than 12 h between two reported locations, no interpolation was done. All interpolated locations that ended up on-land were removed.

#### Determination of metrics and habitat classes

Two movement metrics were calculated for hourly locations: the horizontal swimming speed (m/s) (i.e. ratio of distance to time between 2 successive locations) and the turning angle (degrees) (i.e. turning angle between the previous, the current and the following locations). Additionally, two spatial metrics were calculated for each hourly location, the distance to the nearest coastline (km) and the distance to the nearest glacier front (km). Depending on the time period (1995–2001 or 2013–2016), these distances were calculated using one or the other land/glaciers shapefile, as described above. Using these two spatial metrics, hourly locations were subsequently separated into four different habitat classes (Glacier-Fronts, Fjords, Coastal and At-Sea). If the distance to the nearest glacier front was less than 5 km, the location was assigned to the Glacier-Fronts habitat class. If the distance to the nearest glacier front was more than 5 km and the location was inside a fjord, the location was assigned to the Fjords habitat class. If the distance to the nearest coast was less than 5 km, and the location was not already assigned to one of the two habitat classes above, the location was assigned to the Coastal habitat class. All remaining locations were assigned to the At-Sea habitat class because they were further than 5 km from the coast. The 5 km threshold was chosen to take into account the combination of Argos and land/glaciers data errors.

### Ice data extraction

Sea ice types (i.e. Fast ice, Open Water (0–10%), Very Open Drift Ice (10–40%), Open Drift Ice (40–70%), Close Drift Ice (70–90%), Very Close Drift Ice (90–100%)) were extracted for each hourly location from daily shapefiles obtained from the Norwegian Meteorological Institute (met.no). These data are derived from a combination of products from Radarsat-2 satellite and METOP (i.e. Meteorological Operational Polar Satellite), NOAA (i.e. National Oceanic and Atmospheric Administration) and MODIS (i.e. Moderate Resolution Imaging Spectroradiometer) and have a spatial resolution of approximately 50 m. If a shapefile for a specific day was not available, the shapefile for the nearest day in time was used. If a location occurred outside an area covered by the sea ice shapefiles, the location was assigned to Open Water.

### Modelling approach

The probability of occupying each of the four habitat classes was investigated separately in relation to the day of the year (the number of days since the 7 July (i.e. the earliest tagging date irrespective of year)) and the time period (1995–2001 or 2013–2016). Each hourly location was characterized by four binomial variables, derived from the habitat classes (Glacier-Fronts, Fjords, Coastal, At-Sea). The value 1 was assigned to the habitat variable where the location occurred, while the other variables were then assigned the value 0. Generalized additive mixed effects models (GAMMs) (‘gamm’ function in the R package ‘mgcv) were used to explore each possible relationship (i.e. one model for each habitat class). Day of the year was included as a smooth term while time period was included as a “by-variable” (i.e. day of the year smooth curves were made for each period). Individual ID was included as both a random effect and as a grouping factor in the temporal autocorrelation structure of the order one (corAR1) term. Model selection and model validation was done using the confidence intervals of the corresponding smooth curves, as recommended by [40].

The two movement metrics (swimming speed and turning angle) were investigated separately in relation to the habitat classes and time periods in order to explore potential differences in movement characteristics. Generalized linear mixed effects models (GLMMs) (‘glmmPQL’ function in the R package ‘MASS’) were used to explore possible relationships. Individual was included as a random effect and as a grouping factor in the temporal autocorrelation structure of the order one (corAR1) term to avoid the effects of the hierarchical structure of the data. The swimming speed was log transformed before modelling to meet model assumptions and the models were fitted with a Gaussian family distribution. The distribution of turning angles was not optimal for modelling with a classic distribution; various distribution types were tested (such as Poisson) before selecting the Gaussian as the best fit. Model selection and model validation took place using *p*-values as recommended by [40].

## Results

### Descriptive statistics

**Fig. 1:**
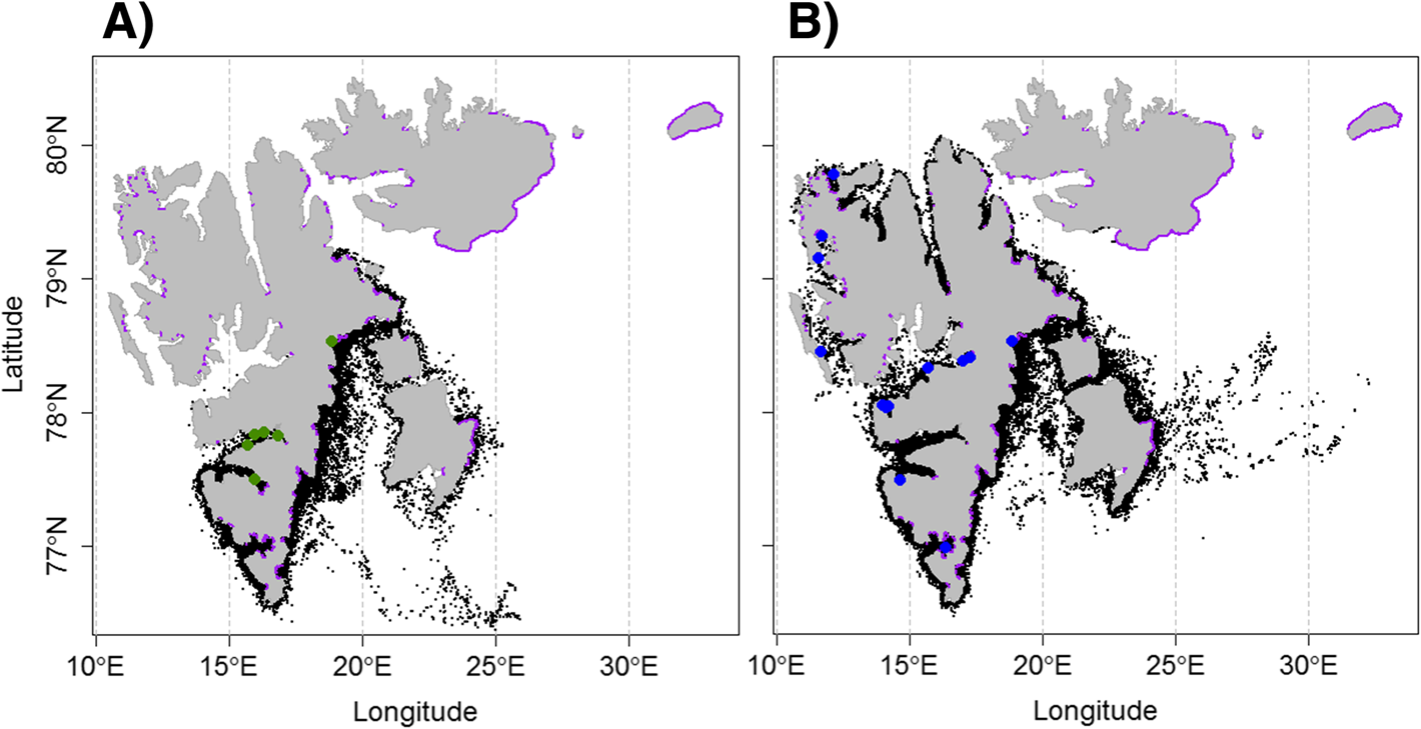
Filtered and corrected tracks of white whales in Svalbard, Norway. Tracks of male white whales equipped with biotelemetry devices in Svalbard, Norway after filtration and correction of on-land positions during **a**) 1995–2001 and **b**) 2013–2016. The blue and green dots represent the deployment points for these two periods. Purple lines correspond to the glacier front data corresponding to the period

The data loggers deployed on the 34 adult male white whales provided location data for periods ranging between 2 and 163 d, with an average duration of 68 ± 45 d (Table 1). The animals from the first period provided data for an average of 51 ± 31 d while animals in the second period provided data for an average of 87 ± 52 d (Table 1). In total, 43,428 locations were reported, including 15,350 locations in the first period and 28,078 in the second period. There were 3422 on-land locations (22% of all the locations) reported in the first period and 6979 on-land locations (25% of all the locations) in the second period. Ninety per cent and 84% of these were corrected successfully, respectively (Fig. 1). After interpolation, 5% (first period) and 12% (second period) of the locations were removed, resulting in a total of 41,410 locations for both time periods combined. Throughout the tracking periods, the white whales generally remained very close to the coastline of Svalbard, with an average distance from shore of 4.90 km (range: 0–117 km) for the 1995–2001 period and 4.33 km from shore (range 0–188 km) for the 2013–2016 period (Fig. 1). The maximum distances from the coastline in both periods occurred when land-fast sea ice “forced” the animals away from the shore. In both periods, the same general seasonal movement pattern was observed with animals moving from the west coast to the east coast around the southern tip of Spitsbergen, as the season progressed (Additional file 2: Figure S2). The white whales spent most of July on the west coast of Spitsbergen. Then, in the period from August to November they occurred both on the west and the east coasts, while during December and January the whales occurred only on the east coast of Spitsbergen (Additional file 2: Figure S2).

**Table 1:** White whales tagging metrics

ID	Tagging date	Tagging location	Tracking duration (days)
1995–3	08.07.1995	77.82–16.85	58
1995–2	09.07.1995	77.85–16.3	30
1995–1	07.07.1995	77.75–15.7	31
1996–1	20.07.1996	77.5–15.97	7
1997–1	04.08.1997	77.83–15.95	113
1997–2	04.08.1997	77.83–15.95	34
1997–3	04.08.1997	77.83–15.95	82
1998–7	01.09.1998	78.53–18.87	7
1999–4	21.08.1999	78.53–18.87	13
1999–3	21.08.1999	78.53–18.87	72
1999–5	18.08.1999	78.53–18.87	63
1999–6	18.08.1999	78.53–18.87	93
1999–7	18.08.1999	78.53–18.87	55
1999–8	19.08.1999	78.53–18.87	65
2000–2	18.10.2000	78.53–18.87	82
2001–1	17.10.2001	78.53–18.87	10
2001–2	18.10.2001	78.53–18.87	62
2001–3	19.10.2001	78.53–18.87	48
2013–1	16.08.2013	79.78–12.16	142
2013–2	23.08.2013	78.41–17.27	96
2013–3	23.08.2013	78.33–15.71	82
2014–8	18.08.2014	77.49–14.66	21
2014–3	14.08.2014	76.98–16.37	127
2014–2	14.08.2014	76.98–16.37	51
2014–5	11.08.2014	76.98–16.37	126
2014–1	11.08.2014	76.98–16.37	20
2014–4	03.08.2014	78.53–18.87	118
2015–5	19.07.2015	79.32–11.72	19
2015–8	19.07.2015	79.15–11.6	2
2016–5	04.08.2016	78.03–14.13	107
2016–3	09.08.2016	78.04–14.22	163
2016–4	19.07.2016	78.45–11.68	56
2016–2	04.08.2016	78.05–14.01	115
2016–1	14.08.2016	78.38–17.03	146

Metrics for 34 male white whales equipped with biotelemetry devices between 1995 and 2016 in Svalbard, Norway, including tagging date, tagging location and tracking duration

**Fig. 2:**
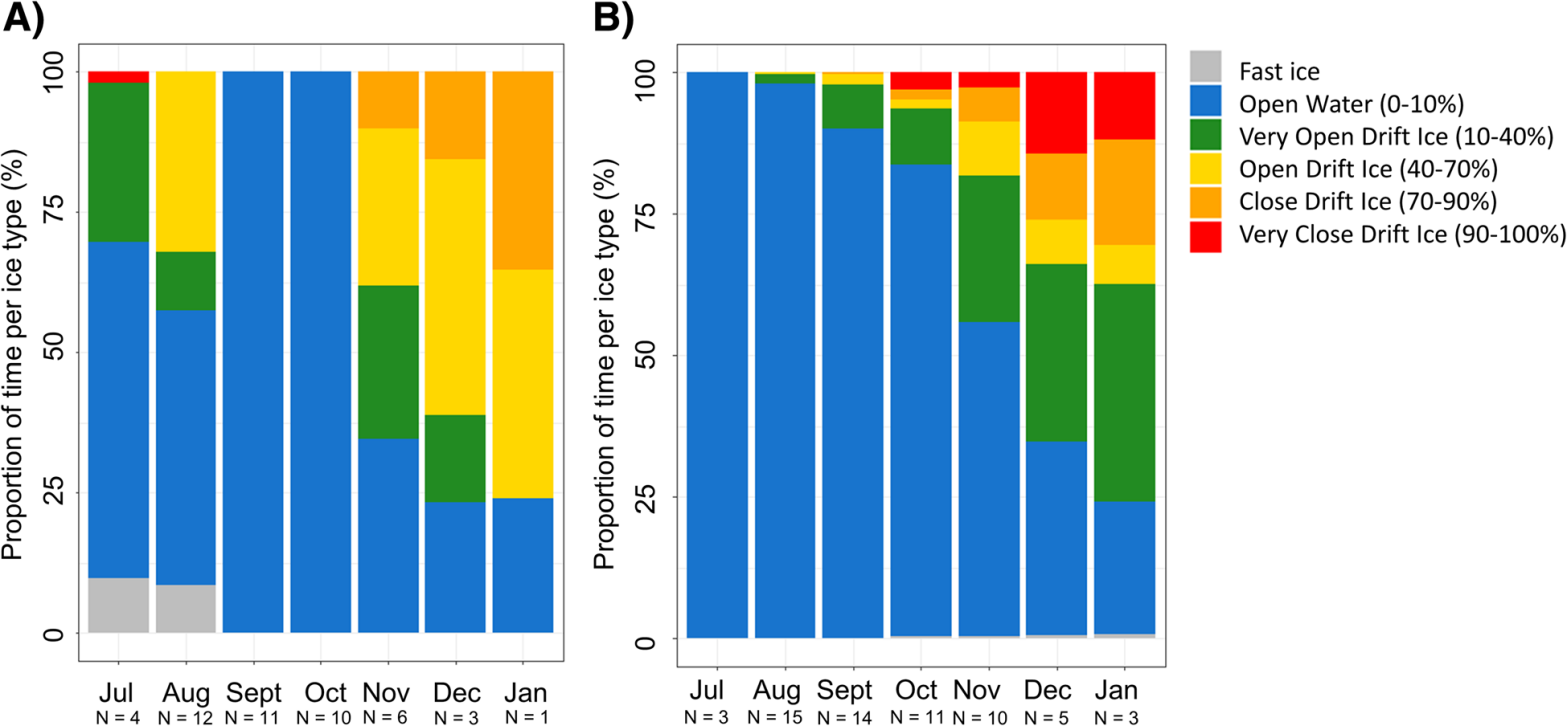
Ice types occupied by white whales, Svalbard, Norway. Graphs **a** and **b** represent the proportion of time spent in each ice type by month by animals of the first time (1995–2001) and second time periods (2013–2016), respectively. N corresponds to the number of individuals being tracked in each month/period

The white whales generally occupied areas with open water or low sea-ice concentrations (less than 10%) from July to October (Fig. 2) during both study periods. One exception from this was that some animals in the first time period occasionally spent time in areas with 10 to 70% sea ice cover and in areas defined as having land-fast ice in July and August. In both periods, the whales occupied areas with ice more frequently from October onward, spending more than 40% of their time in areas with more than 10% sea ice. The whales occasionally also used areas with more than 90% ice cover (Fig. 2), especially in the more recent period.

### Habitat classes

Tracking data from all individuals and both periods, show that the white whales spent 41%, 21%, 24% and 14% of their time in the Glacier-Fronts, Fjords, Coastal and At-Sea habitats, respectively. However, time spent in the various habitats differed between the time periods, with time spent in the Glacier-Fronts habitat declining (from 51% in the first period to 36% in the second) and time spent in the Fjords habitat increasing (from 10 to 26%), while time spent in the other two habitats remained stable (Coastal early 23% vs recent 25%; At-Sea early 16% vs recent 13%).

**Fig. 3:**
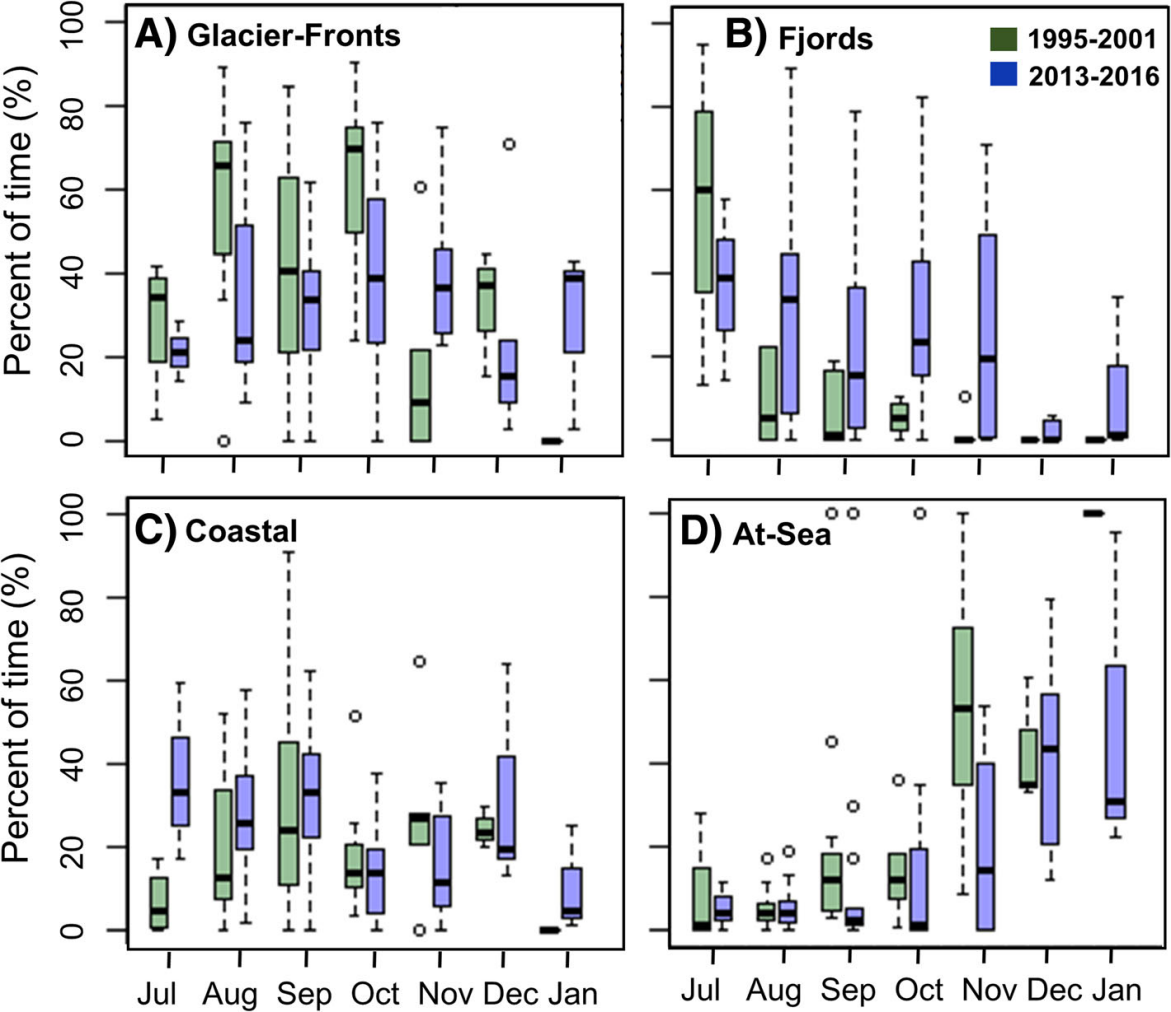
Proportion of time spent in the four habitat classes throughout the first and the second tracking periods. Graphs **a**, **b**, **c** and **d** represent the box plots of proportion of time spent per month for the tracked individuals (see Table 1 for sample size) in the Glacier-Fronts, Fjords, Coastal and the At-Sea habitat classes during the first (green) and the second (blue) time periods

**Fig. 4:**
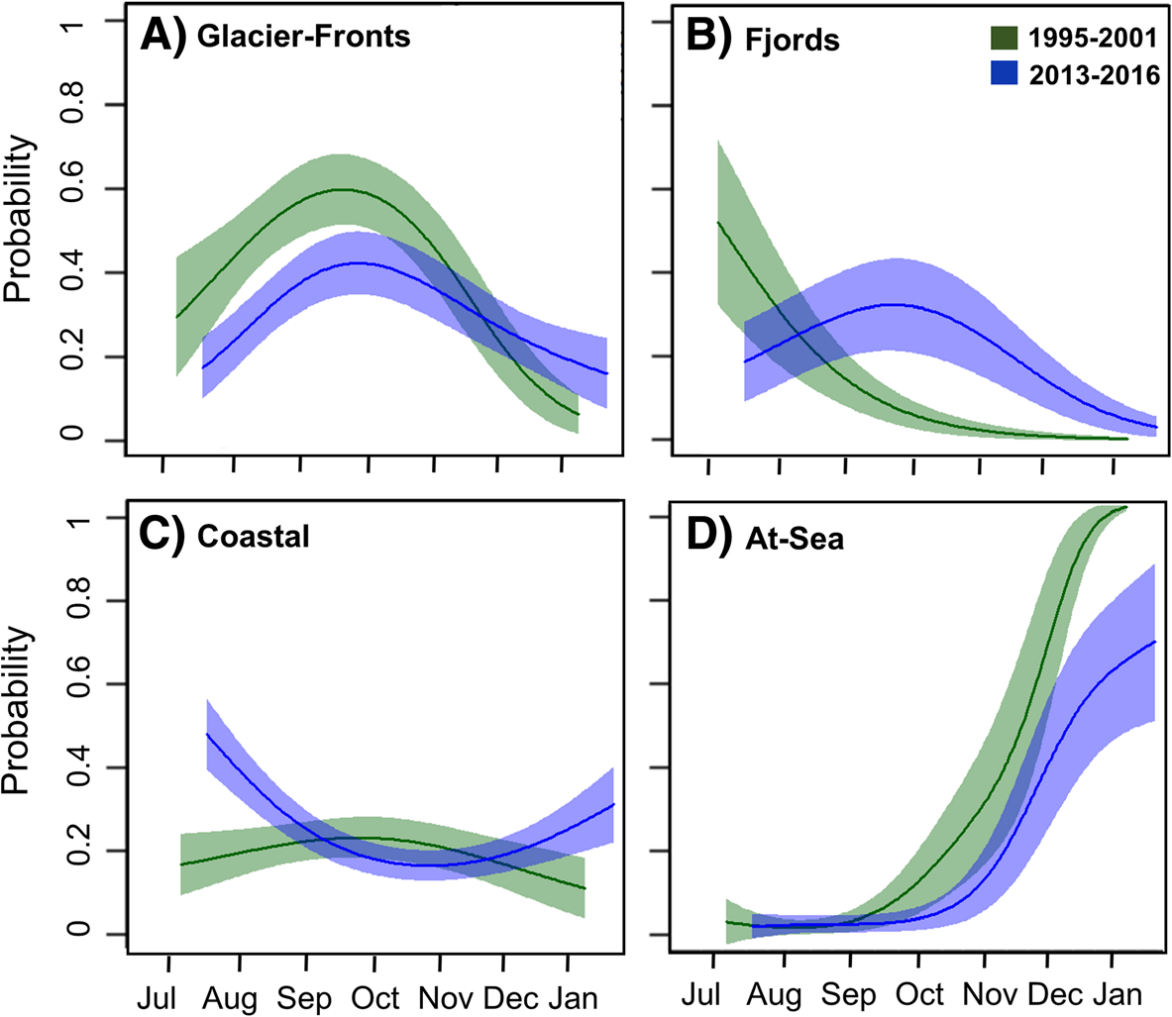
Probability of white whales occurring in each of four habitat classes by day of year. Results of generalized additive mixed effects models showing the probability of white whales being in Glacier-Fronts (**a**), Fjords (**b**), Coastal (**c**) or At-Sea (**d**) habitat classes in Svalbard, Norway, according to day of the year during the first (1995–2001 -green) and the second (2013–2016 - blue) time periods. Values shown are mean ± 95% CI

Seasonal variation in the proportion of time spent in each habitat class throughout the tracking periods is shown in Fig. 3. In both periods, the whales spent most of their time in the Glacier-Fronts habitat, especially towards the end of the summer and in the early autumn (Fig. 3a). As winter progressed animals were pushed into the At-Sea habitat by the formation of ice in both periods (Fig. 3d). Results of GAMMs investigating the probability of being in a given habitat class, depending on day of the year and the time period are represented in Fig. 4. For each habitat class, the best model included both the period and the day of the year, as well as their interactions. The probability of being in the Glacier-Fronts habitat was higher at the end of the summer and at the beginning of the autumn compared to other seasons, and animals had a higher probability of being in the Glacier-Fronts habitat in the first period than in the second period (Fig. 4a). The probability of occupying the Fjords habitat was higher in the second period than in the first period, except in July (Fig. 4b). The whales had a low probability of being in the Coastal habitat, though they spent more time in this habitat in summer and in winter in the second period (Fig. 4c). Finally, the animals had a low probability of being in the At-Sea habitat during summer and early autumn, but the probability of being in the At-Sea habitat increased exponentially to more than 0.6 at the end of the autumn and during the winter (Fig. 4d). Animals from the first period entered the At-Sea habitat earlier in the year than during the second period (Fig. 4d). It is important to note that animals moved over much larger areas within Svalbard in the second period compared to animals tracked during the first period. This difference could in part be due to an effect of the deployment sites, which were more widely geographically distributed around the archipelago in the second period. To explore this potential biases further, the analyses were re-run excluding part of the tracks from the second period that were from areas not visited by whales during the first period (Additional file 3: Figure S3). This analysis gave the same results for space use patterns between the first and the second periods (Additional file 4: Figure S4).

### Movement patterns

**Fig. 5:**
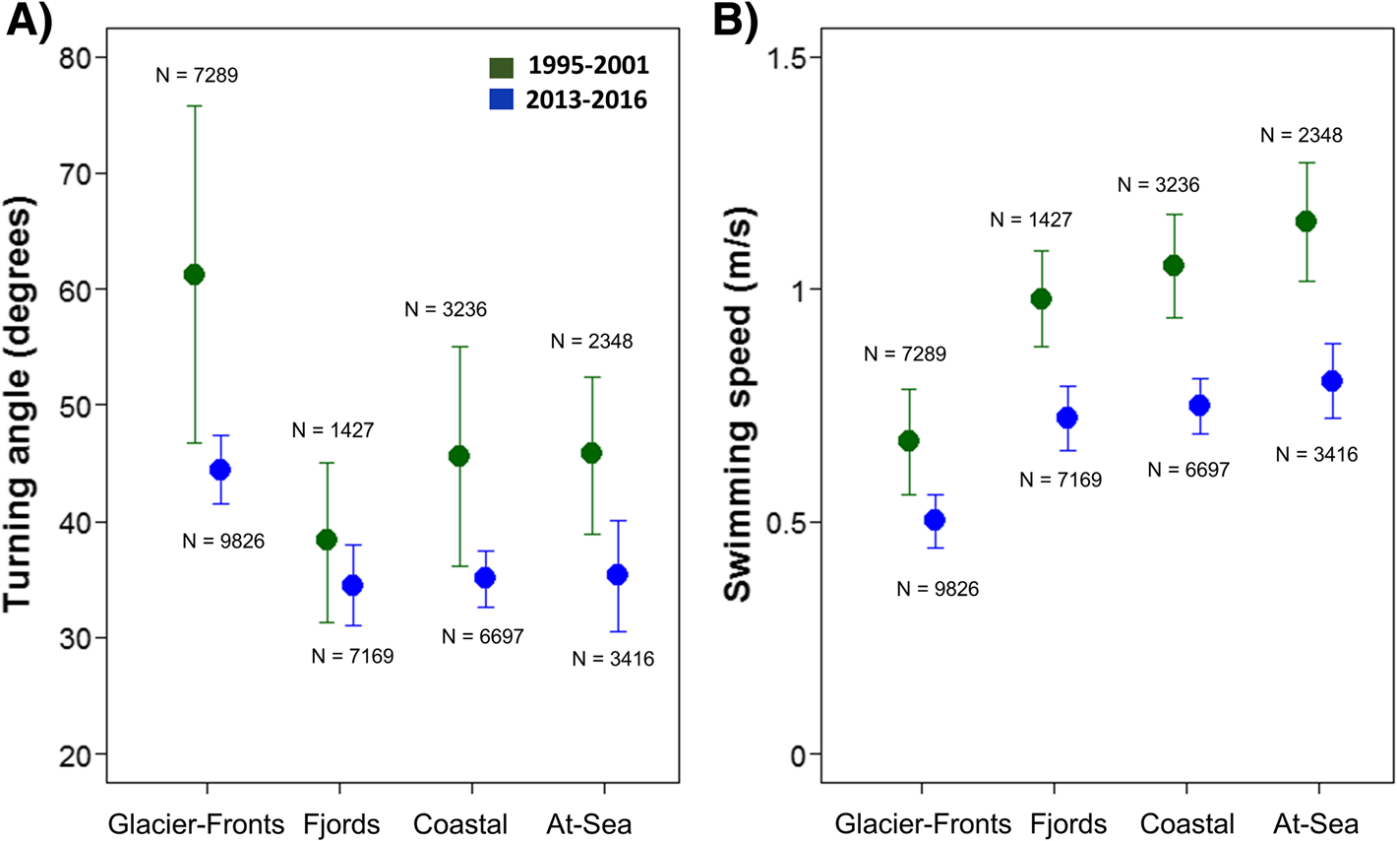
Bootstrapping 95% confidence intervals (CI) of turning angles (degrees) and swimming speeds (m/s) by white whales in the four habitat classes for the two tracking periods. Graphs **a** and **b** represent turning angles (degrees) and swimming speeds (m/s) of individual white whales during the first (1995–2001 -green) and the second (2013–2016 -blue) time periods. The number of hourly locations within each category is displayed above or below each CI

The average swimming speed of the white whales was 0.81 ± 0.63 m/s (mean ± SD) for the first and 0.66 ± 0.65 m/s for the second period, respectively. Their movements were characterized by an average turning angle of 55.39 ± 64.40 degrees in the first and 39.68 ± 59.28 degrees in the more recent period. The movement metrics for the different habitat classes for each time period are summarized in Fig. 5. Results of generalized linear mixed effects models investigating the relationships between the movement metrics, habitat classes and periods are presented in the Tables 2 and 3. The best model for each metric included both the time period and the habitat classes as well as their interactions, highlighting that the turning angle and the swimming speed differ between the habitat classes and the time periods. The whales had higher turning angles and swimming speeds in the first period compared to the second period. Animals from both periods increased their turning angles and decreased their swimming speeds in the Glacier-Fronts habitat in comparison to the other three habitat classes, where they seem to behave quite consistently (Fig. 5, Tables 2 and 3). However, the differences between both movement metrics in the Glacier-Fronts and the Fjords habitats was reduced in the second time period compared with the first (Fig. 5, Tables 2 and Table 3).

**Table 2:** Turning angles of white whales equipped with satellite transmitters in Svalbard, Norway, as a function of habitat class and time period

Model parameter(s)	Value	Std error	*P* value
(Intercept)	5.82E + 01	2.06E + 00	0.00E + 00
habitatCoastal	−1.08E + 01	1.47E + 00	0.00E + 00
habitatFjords	−1.79E + 01	2.09E + 00	0.00E + 00
habitatAt-sea	-1.41E + 01	1.67E + 00	0.00E + 00
period2013–2016	-1.31E + 01	2.87E + 00	0.00E + 00
period2013–2016: habitatCoastal	1.04E + 00	1.85E + 00	5.70E-01
period2013–2016: habitatFjords	8.35E + 00	2.39E + 00	0.00E + 00
period2013–2016: habitatAt-sea	2.76E + 00	2.16E + 00	2.00E-01

Results of generalized linear mixed effects models showing the effect of the habitat class and time period on the turning angle of white whales in Svalbard, Norway

**Table 3:** Swimming speed of white whales equipped with satellite transmitters in Svalbard, Norway, as a function of habitat class and time period

Model parameter(s)	Value	Std error	*P* value
(Intercept)	−7.50E-01	5.30E-02	0.00E + 00
habitatCoastal	2.80E-01	2.55E-02	0.00E + 00
habitatFjords	3.00E-01	4.17E-02	0.00E + 00
habitatAt-sea	3.50E-01	3.21E-02	0.00E + 00
period2013–2016	−3.40E-01	7.47E-02	0.00E + 00
period2013–2016: habitatCoastal	−2.00E-03	3.26E-02	9.41E-01
period2013–2016: habitatFjords	−9.70E-02	4.83E-02	4.46E-02
period2013–2016: habitatAt-sea	4.20E-02	4.35E-02	3.27E-01

Results of generalized linear mixed effects models showing the effect of habitat class and period on the swimming speed of white whales in Svalbard, Norway

## Discussion

The Svalbard area has experienced extreme increases in both air and sea temperatures in recent decades, resulting in a dramatic decline in sea ice cover and massive retractions of tidal glaciers [2, 9, 15, 16]. In the present study, a unique biotelemetry data set spanning over 20 years was used to investigate possible responses to these significant habitat changes for one of Svalbard’s resident ice-associated marine mammal species that has a particularly strong affiliations with glacier fronts - the white whale. The fact that white whales in Svalbard have extremely coastal movement patterns, combined with Argos location errors (which were somewhat different in the two periods), in addition to the continuous change of land/glacier positions, created some analytical challenges. Many locations were registered as being on land (or on glaciers). To overcome this problem a separate set of land/glacier data was employed for the two time periods that took into account the change in geographical positions of the glacier fronts over time. In addition, all of the on-land locations were corrected, such that they were shifted to being the sea. Although this process might introduce some bias (only on-land locations corrected and not the at-sea locations), this species is strictly marine, so on-land positions were by definition impossible, and therefore the correction was deemed appropriate. Shifting the on-land locations to positions at sea by using only their possible “at sea particles” (probable locations) likely resulted in the whale locations being “pushed” further offshore than they really were. In response to these corrections and also to deal with the uncertainty of the “real” at sea locations (as well as the uncertainty of the land/glaciers data), quite large thresholds (5 km) were used to separate the various habitat classes, which likely compensates somewhat for potential errors in classification of the habitat classes. This 5 km threshold was also used to minimize any potential biases introduced by the differences in the Argos errors between the two periods. Thus, even though more precise locations (i.e. Global Positioning System (GPS)), as well as more precise land/glaciers data (i.e. annual positions), would have been preferable, the results presented here are thought to be quite reliable, allowing meaningful comparisons between the two periods.

White whales in Svalbard remain close to the coast year round, with more locations on the west coast in summer and more locations in the east coast in winter. This tightly coastal distribution was documented previously by [25] in the late 1990s and early 2000s, and clearly from the data obtained in the recent tracking period, continues to be the case. The behaviour of this population is striking when compared to movement behaviours of others populations, many of which move long distances offshore (i.e. [21]). Additionally, similar to the previous findings of [25], the present study also documented that the white whales in Svalbard continue to spend most of their time close to glacier fronts, although the time spent in this habitat has declined from 51% of their time to 36% of their time between the two study periods. The close affiliation to glacier fronts has also been confirmed by a recent study based on the analyses of 13 years of cetacean sighting data (2002–2014) from waters around the Svalbard Archipelago, in which white whales were observed only in near shore habitats, often in areas containing sea ice and having somewhat lower SSTs than areas occupied by seasonally resident species [30].

The probability of being in a particular habitat class varied seasonally as well as between the two tracking periods. The whales were most tightly affiliated with glacier fronts in the late summer and autumn and were found to spend less time in front of glacier fronts in the second time period than in the first one. In contrast, animals were found to spend more time in the fjords in the second period, mainly in autumn, compared to the first period. Animals from both periods spent little time in Coastal habitats (outside fjords), except during the summer and the end of the winter in recent years, when animals spent more time in this habitat than the animals from the first period. Finally, in winter, the whales dispersed into the At-Sea habitat; this occurred later in the year and more pronouncedly in the second period. Winter sample sizes are however small in both periods, so these suggested patterns must be interpreted with caution.

The white whales had the highest turning angles and the lowest swimming speeds in the Glacier-Fronts habitat. This was the case in both tracking periods. This sort of area-restricted search (ARS) behaviour is often associated with foraging [41, 42]. This result is thus in accordance with the general knowledge that tidal glacier fronts represent hotspots for foraging for many species [2]. Fishes in the Svalbard area that could be attracted to cold, high-productivity areas such as glacial fronts include schooling fishes such as polar cod and capelin*.* As said previously, these fish species are known to be favoured prey of white whales in other regions [43, 44] and in Svalbard [31]. In addition, tidal glacier fronts, which are known to also be associated with large outflows of freshwater, could be of interest for white whales for their moulting process too. It has been suggested that movements into fresh water may enhance shedding of the cork and skin layers in these whales [45, 46]. However, moulting activities mainly take place in the summer and the white whales in Svalbard spend most of their time in the autumn in these areas as well, reinforcing the assumption that these areas represent important foraging areas. It is worth noting that the swimming speeds, as well as the turning angles, calculated in this study are minimum speeds and angles. The positions of the hourly locations that formed the basis for these calculations were interpolated as straight lines between transmitted positions, while the whales undoubtedly deviated from such linear behaviour.

The question arises as to why white whales in Svalbard spend less time in the Glacier-Fronts habitat in recent years if these areas constitute important foraging areas. The likely reason is that there have been dramatic changes in the hydrographic conditions in Svalbard, especially on the west coast between the two tracking periods. Atlantic water, with higher temperatures than anything previously recorded in Svalbard [47] has recently become much more common in the fjords of west Spitsbergen. This has caused reduced ice formation and has allowed for influxes of more temperate and boreal fish species [47–49]. Fish species such as Atlantic cod (*Gadus morhua*), haddock (*Melanogrammus aeglefinus*) and herring (*Clupea harengus*) have recently replaced the native Arctic fish fauna to a large degree, in particular polar cod (*Boreogadus saida*) has declined in the region [50]. The appearance of these pelagic and benthic fishes, which are not affiliated with glaciers, likely explains the shift by the white whales towards spending more time in the Fjords habitat during the recent tracking period. However, animals continued to spend a lot of time in front of glacier fronts in the recent period, so the increased time spent in Fjord habitat might be due to them targeting a new, additional source of food rather than being a strict dietary change. It is important to specify that no diet analyses are included in the present study, so the suggested feeding behaviour in Fjord habitat is speculative. However, such dietary shifting, or at least a generalization of the diet, has been documented recently in white whales in other locations, e.g. Cumberland Sound, where white whales have changed their summer diet from Arctic cod to capelin in recent years [29]. The suggestion of some feeding taking place away from glacier fronts in the recent period is supported by the movement metrics analyses in the present study. The difference in both swimming speed and turning angles between the Glacier-Fronts and the Fjords habitats were weaker in the second time period compared with the first, suggesting that the Fjords habitat was likely used more as a foraging habitat during the second period. This suggestion does however need to be viewed with some caution, because the locations data from the second period are more precise and more frequent than in the first period, introducing potential biases in the interpretation of speed and turning angles.

The low amount of time spent in Coastal areas throughout both study periods suggests that this habitat is likely only used as a transit corridor between areas of interest to the whales. One reason for travelling along the coast could simply be that following the shoreline is the shortest distance between preferred foraging habitats, i.e. Glacier-Fronts and/or Fjords. Another reason could be associated with anti-predator behaviour [51], especially avoidance of killer whales*, Orcinus orca*, which are known to prey on white whales [52, 53]. In response to decreasing sea ice and increasing Atlantic water inflow, a northward expansion of seasonally occurring cetaceans is expected [26, 30]. In Svalbard, killer whales are observed regularly, but not frequently enough to explore time trends [30]. However, in other places in the Arctic, such as the eastern Canadian Arctic, killer whale sightings have increased exponentially since 1900 [54]. The extremely coastal movement of the Svalbard white whales is likely a result of a combination of minimizing travelling time and avoiding predation. Such an explanation is also supported by the fact that white whales in Svalbard are unusually quiet (few vocalisations) compared to white whales from other areas [55].

In the winter, white whales occurred more frequently in the At-Sea habitat (at more than 5 km to the coast) during both tracking periods and they spent longer periods in ice-covered waters in this season. When sea ice forms along the coast, the whales are forced to leave the shore and move into areas with drifting ice, where it is easy for them to surface to breathe, and since sea ice has formed later during the second time period, the dispersal from the coast naturally takes place later in the years in the recent period. It might be the ice itself that acts as the trigger to stimulate movement offshore, but it is also possible that associated environmental features might play a role [56]. Found that white whale migrations in Baffin Bay were correlated with sea surface temperature, though this variable is also likely to be correlated with seasonal progression. It is important to note that the delay in ice formation in Svalbard in the second period compared to the first period automatically allows the animals to remain in the other habitats for a longer period before moving offshore into ice-covered waters. This habitat is quite normal for white whales, and it likely provides several advantages, including shelter from open-water predators and from inclement weather that is more common in winter [3, 4]. Occasionally, even during summer (in the first time period), white whale locations close to the coast were detected in relative high concentrations of sea ice and even in land-fast ice areas. This is consistent with the findings of [30], in which 25% of the recorded observations of white whales around the Svalbard Archipelago occurred in areas with at least 30% ice cover.

It is important to note that only males were included in the present study and hence all the results presented here describe only a change in space use patterns for this sex. This bias toward males in this study was due to selection of large, white animals for tagging. In this sexually dimorphic species, males get bigger and females stay grey longer (in some cases well beyond the age of sexual maturity). Additionally, capture nets were pulled whenever young (grey-brown) calves were detected in groups, thus biasing captures toward all male groups. The sex ratio of the population is probably normal (1:1), but we believe that the sampling decisions before and after capture biased the tagged sample (i.e. 34 males among 38 equipped individuals). Movement and habitat patterns of female white whales in Svalbard require future research attention.

## Conclusions

In this study, a unique biotelemetry data set spanning 20 + years was used to investigate the impacts of the environmental changes that have occurred in the Svalbard Archipelago during recent decades on the space use patterns of white whales. Comparing periods before and after a collapse in sea ice that occurred in 2006, which has persisted since, marked changes were detected. Following the decline in sea ice, white whales now spend less time near glacier fronts and more time out in the fjords. This habitat change suggests that a shift in diet or at least a generalization of the diet (animals still spend high amount of time in front of glacier fronts) has occurred in Svalbard, away from only Arctic fish species and towards more Atlantic fishes, as has been observed in other places within the white whale’s range. White whales continue to spend little time in coastal habitats outside fjords as they move around the archipelago. This probably reflects optimal routing between foraging areas and perhaps also avoidance of open water predators. Finally, animals spent the majority of their time during the winter in drifting offshore sea ice; a habitat that provides shelter both against predators and inclement weather and also provides easy access to the surface for breathing, which the inshore areas may not in this season. The change in space use patterns documented between the two periods in this study suggests that white whales, at least in the short-term, are resilient enough in their habitat preferences to allow them to adapt to an Arctic with less sea ice coverage.

## Additional files

Additional file 1: **Figure S1.** Photographs showing satellite tags used on white whales in A) the first time period (1995–2001) and B) the second time period (2013–2016), Svalbard, Norway. (TIF 2373 kb)

Additional file 2: **Figure S2.** Movements of white whales throughout the first (1995–2001) and the second (2013–2016) tracking periods. Hourly locations of the 34 male white whales tracked in Svalbard, Norway, after the interpolation of the filtered and on-land corrected tracks per month. A) represents the movement of animals throughout the first tracking period (1995–2001) and B) represents the movement of animals throughout the second tracking period (2013–2016). N corresponds to the number of individuals reporting data per month. Purple lines correspond to the tidal glacier front data corresponding to each of the two periods. (TIF 2847 kb)

Additional file 3: **Figure S3.** Filtered and corrected tracks for white whales satellite tagged in Svalbard, Norway, during two time periods (excluding the non-common areas between the two periods). Tracks of 34 male white whales equipped with biotelemetry devices in Svalbard, Norway after filtration and correction of on-land positions during A) the period 1995–2001 and B) the period 2013–2016. The blue and green dots represent the deployment points for these two periods. Purple lines correspond to the tidal glacier front data corresponding to each of the periods. (TIF 1923 kb)

Additional file 4: **Figure S4.** Probability of white whales being in each of four habitat classes by day of year, excluding the non-common areas between the two periods. Results of generalized additive mixed effects models showing the probability of white whales being in Glacier-Front (A), Fjord (B), Coastal (C) or At-Sea (D) habitat classes in Svalbard, Norway, according to day of the year during the first (1995–2001 -green) and the second (2013–2016 - blue) time periods. Values shown are mean ± 95% CI. (TIF 1035 kb)

## Abbreviations

ARS: Area Restricted Search
CTD-SRDLs: Conductivity-Temperature-Depth Satellite Relay Data Loggers
GAMMs: Generalized Additive Mixed effects Models
GLMMs: Generalized Linear Mixed effects Models
GPS: Global Positioning System
IUCN: International Union for Conservation of Nature
LC: Location Class
METOP: Meteorological Operational Polar Satellite
MODIS: Moderate Resolution Imaging Spectroradiometer
NOAA: National Oceanic and Atmospheric Administration
NPI: Norwegian Polar Institute
SDA: Speed Distance and Angle
SRDLs: Satellite Relay Data Loggers
WSC: West Spitsbergen Current

## References

[1] Stroeve J, Holland MM, Meier W, Scambos T, Serreze M. Arctic Sea ice decline: faster than forecast. Geophys Res Lett. 2007. 10.1029/2007GL029703.

[2] Lydersen C, Assmy P, Falk-Petersen S, Kohler J, Kovacs KM, Reigstad M (2014). The importance of tidewater glaciers for marine mammals and seabirds in Svalbard, Norway. J Mar Syst.

[3] Stirling I (1997). The importance of polynyas, ice edges, and leads to marine mammals and birds. J Mar Syst.

[4] Heide-Jørgensen MP, Laidre KL (2004). Declining extent of open-water Refugia for top predators in Baffin Bay and adjacent waters. Ambio.

[5] Laidre KL, Stern H, Kovacs KM, Lowry L, Moore SE, Regehr EV (2015). Arctic marine mammal population status, sea ice habitat loss, and conservation recommendations for the 21^st^ century. Conserv Biol.

[6] Pavlov Alexey K., Tverberg Vigdis, Ivanov Boris V., Nilsen Frank, Falk-Petersen Stig, Granskog Mats A. (2013). Warming of Atlantic Water in two west Spitsbergen fjords over the last century (1912–2009). Polar Research.

[7] Nordli Øyvind, Przybylak Rajmund, Ogilvie Astrid E.J., Isaksen Ketil (2014). Long-term temperature trends and variability on Spitsbergen: the extended Svalbard Airport temperature series, 1898–2012. Polar Research.

[8] Onarheim Ingrid H., Smedsrud Lars H., Ingvaldsen Randi B., Nilsen Frank (2014). Loss of sea ice during winter north of Svalbard. Tellus A: Dynamic Meteorology and Oceanography.

[9] Muckenhuber S, Nilsen F, Korosov A, Sandven S (2016). Sea ice cover in Isfjorden and Hornsund, Svalbard (2000-2014) from remote sensing data. Cryosphere.

[10] Wiencke C, Hop H (2016). Ecosystem Kongsfjorden: new views after more than a decade of research. Polar Biol.

[11] Dowdeswell JA, Hagen JO, Bjornsson H, Glazovsky AF, Harrison WD, Holmlund P (1997). The mass balance of circum-Arctic glaciers and recent climate change. Quat Res.

[12] Hagen JO, Kohler J, Melvold K, Winther JG (2003). Glaciers in Svalbard: mass balance, runoff and freshwater flux. Polar Res.

[13] Hagen JO, Melvold K, Pinglot F, Dowdeswell JA (2003). On the net mass balance of the glaciers and ice caps in Svalbard, Norwegian Arctic. Arct Antarct Alp Res.

[14] Wouters B, Chambers D, Schrama EJO. GRACE observes small-scale mass loss in Greenland. Geophys Res Lett. 2008. 10.1029/2008GL034816.

[15] Moholdt G, Hagen JO, Eiken T, Schuler TV (2010). Geometric changes and mass balance of the Austfonna ice cap, Svalbard. Cryosphere.

[16] Nuth C, Moholdt G, Kohler J, Hagen JO, Kääb A. Svalbard glacier elevation changes and contribution to sea level rise. J Geophys Res. 2010. 10.1029/2008JF001223.

[17] Kovacs KM, Lydersen C (2008). Climate change impacts on seals and whales in the North Atlantic Arctic and adjacent shelf seas. Sci Progr.

[18] Lowry L, Reeves R, Laidre K. Delphinapterus leucas. The IUCN Red List of Threatened Species. 2017. 10.2305/IUCN.UK.2017-3.RLTS.T6335A50352346.en.

[19] Martin AR, Smith TG, Cox OP (1993). Studying the behaviour and movements of high Arctic belugas with satellite telemetry. Symp Zool Soc Lond.

[20] Smith TG, Martin AR (1994). Distribution of belugas, *Delphinapterus leucas,* in the Canadian high Arctic. Can J Fish Aquat Sci.

[21] Richard PR, Martin A, Orr JR (1997). Study of summer and fall movements and dive behaviour of Beaufort Sea belugas, using satellite telemetry: 1992–1995. Environ stud res funds rep 134.Calgary.

[22] Lesage V, Kingsley MCS (1998). Updated status of the St. Lawrence River population of the beluga, *Delphinapterus leucas*. Can Field Nat.

[23] Richard PR, Heide-Jörgensen MP, St Aubin DJ (1998). Fall movements of belugas *(Delphinapterus leucas)* with satellite- linked transmitters in Lancaster sound, Jones sound, and northern Baffin Bay. Arctic.

[24] Richard PR, Martin A, Orr JR (2001). Summer and autumn movements of belugas of the eastern Beaufort Sea stock. Arctic.

[25] Lydersen C, Martin AR, Kovacs KM, Gjertz I (2001). Summer and autumn movements of white whales *Delphinapterus leucas* in Svalbard, Norway. Mar Ecol Prog Ser.

[26] Reeves RR, Ewins PJ, Agbayani S, Heide-Jørgensen MP, Kovacs KM, Lydersen C (2014). Distribution of endemic cetaceans in relation to hydrocarbon development and commercial shipping in a warming Arctic. Mar Policy.

[27] Hauser Donna D. W., Laidre Kristin L., Stern Harry L., Moore Sue E., Suydam Robert S., Richard Pierre R. (2017). Habitat selection by two beluga whale populations in the Chukchi and Beaufort seas. PLOS ONE.

[28] Moore SE, Shelden KEW, Litzky LK, Mahoney BA, Rugh DJ (2000). Beluga whale, *Delphinapterus leucas*, habitat associations in cook inlet. Alaska Mar Fish Rev.

[29] Watt CA, Orr J, Ferguson SH (2016). A shift in foraging behaviour of beluga whales *Delphinapterus leucas* from the threatened Cumberland sound population may reflect a changing Arctic food web. Endang Species Res.

[30] Storrie Luke, Lydersen Christian, Andersen Magnus, Wynn Russell B., Kovacs Kit M. (2018). Determining the species assemblage and habitat use of cetaceans in the Svalbard Archipelago, based on observations from 2002 to 2014. Polar Research.

[31] Dahl TM, Lydersen C, Kovacs KM, Falk-Petersen S, Sargent J, Gjertz I (2000). Fatty acid composition of the blubber in white whales *(Delphinapterus leucas)*. Polar Biol.

[32] Brodie PF (1971). A reconsideration of aspects of growth, reproduction, and behavior of white whale *(Delphinapterus leucas),* with reference to the Cumberland sound, Baffin Island, population. J Fish Res Board Can.

[33] Fedak M, Lovell P, McConnell B, Hunter C. Methods for overcoming the constraints of long range telemetry of biological information from animals: getting more useful data from small packages. Am Zool 2000;40:1013–1013.

[34] Freitas C, Lydersen C, Fedak MA, Kovacs KM (2008). A simple new algorithm to filter marine mammal Argos locations. Mar Mamm Sci.

[35] Freitas C. Package `Argosfilter'. 2013. https://cran.rproject.org/web/packages/argosfilter/argosfilter.pdf.

[36] Tremblay Y, Robinson PW, Costa DP. A parsimonious approach to modeling animal movement data. PLoS One. 2009. 10.1371/%20journal.pone.0004711. 10.1371/journal.pone.0004711 PMC2650804 19262755

[37] König M, Nuth C, Kohler J, Moholdt G, Pettersen R, Kargel JS, Leonard GJ, Bishop MP, Kääb A, Raup BH (2013). A digital glacier database for Svalbard. Global land ice measurements from space: satellite multispectral imaging of glaciers.

[38] Costa Daniel P., Robinson Patrick W., Arnould John P. Y., Harrison Autumn-Lynn, Simmons Samantha E., Hassrick Jason L., Hoskins Andrew J., Kirkman Stephen P., Oosthuizen Herman, Villegas-Amtmann Stella, Crocker Daniel E. (2010). Accuracy of ARGOS Locations of Pinnipeds at-Sea Estimated Using Fastloc GPS. PLoS ONE.

[39] Lowther Andrew D., Lydersen Christian, Fedak Mike A., Lovell Phil, Kovacs Kit M. (2015). The Argos-CLS Kalman Filter: Error Structures and State-Space Modelling Relative to Fastloc GPS Data. PLOS ONE.

[40] Zuur AF, Ieno EN, Walker NJ, Saveliev AA, Smith GM (2009). Mixed effects models and extensions in ecology with R.

[41] Charnov EL (1976). Optimal foraging, the marginal value theorem. Theor Popul Biol.

[42] Benhamou S, Bovet P (1989). How animals use their environment: a new look at kinesis. Anim Behav.

[43] Seaman GA, Lowry LF, Frost KJ (1982). Foods of belukha whales (*Delphinapterus leucas*) in western Alaska. Cetology.

[44] Boltunov AN, Belikov SE (2002). Beluga (*Delphinapterus leucas*) of the Barents, Kara and Laptev seas. In Heide-Jørgensen MP, Wiig Ø, editors Belugas in the North Atlantic and the Russian Arctic NAMMCO Sci Publ.

[45] St Aubin DJ, Smith TG, Geraci JR (1990). Seasonal epidermal moult in beluga whales, *Delphinapterus leucas*. Can J Zool.

[46] Boily P (1995). Theoretical heat flux in water and habitat selection of phocid seals and beluga whales during annual moult. J Theor Biol.

[47] Spielhagen RF, Werner K, Sørensen SA, Zamelczyk K, Kandiano E, Budeus G (2011). Enhanced modern heat transfer to the Arctic by warm Atlantic water. Science.

[48] Cottier FR, Nilsen F, Inal ME, Gerland S, Tverberg V, Svendsen H. Wintertime warming of an Arctic shelf in response to large-scale atmospheric circulation. Geophys Res Lett. 2007. 10.1029/2007GL029948.

[49] Berge J, Heggland K, Lonne OJ, Cottier F, Hop H, Gabrielsen GW (2015). First records of Atlantic mackerel (*Scomber scombrus*) from the Svalbard archipelago, Norway, with possible explanations for the extension of its distribution. Arctic.

[50] Fossheim Maria, Primicerio Raul, Johannesen Edda, Ingvaldsen Randi B., Aschan Michaela M., Dolgov Andrey V. (2015). Recent warming leads to a rapid borealization of fish communities in the Arctic. Nature Climate Change.

[51] Finley KJ, Miller GW, Davis RA, Green CR (1990). Reactions of belugas *Delphinapterus leucas* and narwhals, *Monodon monoceros,* to ice-breaking ships in the Canadian high arctic. Can Bull Fish Aquat Sci.

[52] Kleinenberg SE, Yablokov AV, Belkovich BM, Tarasevich MN (1969). Beluga *(Delphinapterus leucas).* Investigation of the species.

[53] George JC, Suydam R (1998). Observations of killer whale *(Orcinus orca)* predation in the northeastern Chukchi and western Beaufort seas. Mar Mamm Sci..

[54] Higdon JW, Ferguson SH (2009). Loss of Arctic Sea ice causing punctuated change in sightings of killer whales (*Orcinus orca*) over the past century. Ecol Appl.

[55] Karlsen JD, Bisther A, Lydersen C, Haug T, Kovacs KM (2002). Summer vocalisations of adult male white whales (*Delphinapterus leucas*) in Svalbard. Norway Polar Biol.

[56] Bailleul F, Lesage F, Power M, Doidge SW, Hammill MO (2012). Migration phenology of beluga whales in a changing Arctic. Clim Res.

