# Supplementary figures and images for: Habitats and movement patterns of white whales Delphinapterus leucas in Svalbard, Norway in a changing climate
Source: Mov Ecol. 2018 Oct 24;6:21. doi: 10.1186/s40462-018-0139-z (PMC6199748; doi:10.1186/s40462-018-0139-z)

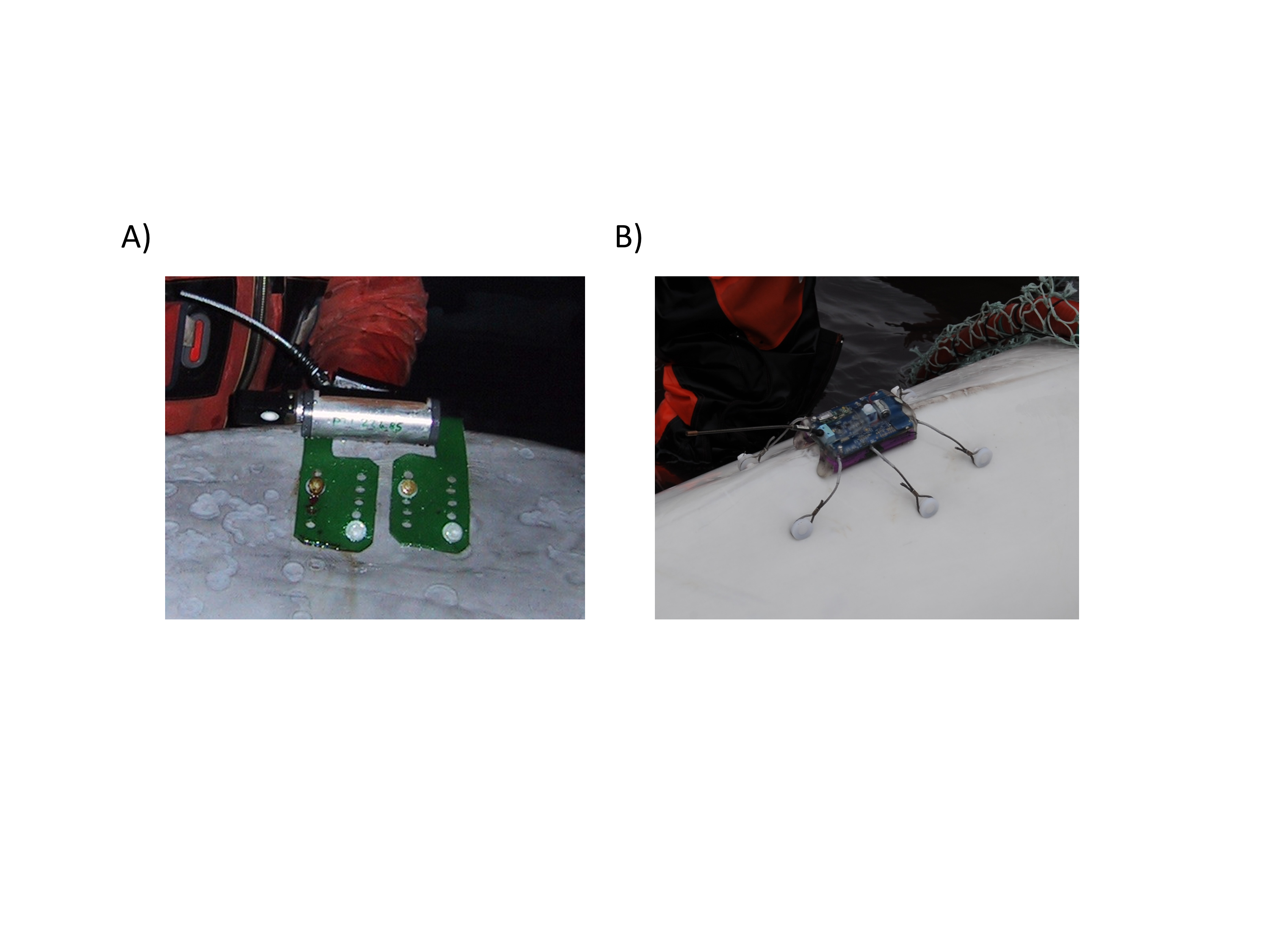

Supplement: Supplementary file 1 — Figure S1. Photographs showing satellite tags used on white whales in A) the first time period (1995–2001) and B) the second time period (2013–2016), Svalbard, Norway. (TIF 2373 kb) [file 40462_2018_139_MOESM1_ESM.tif]

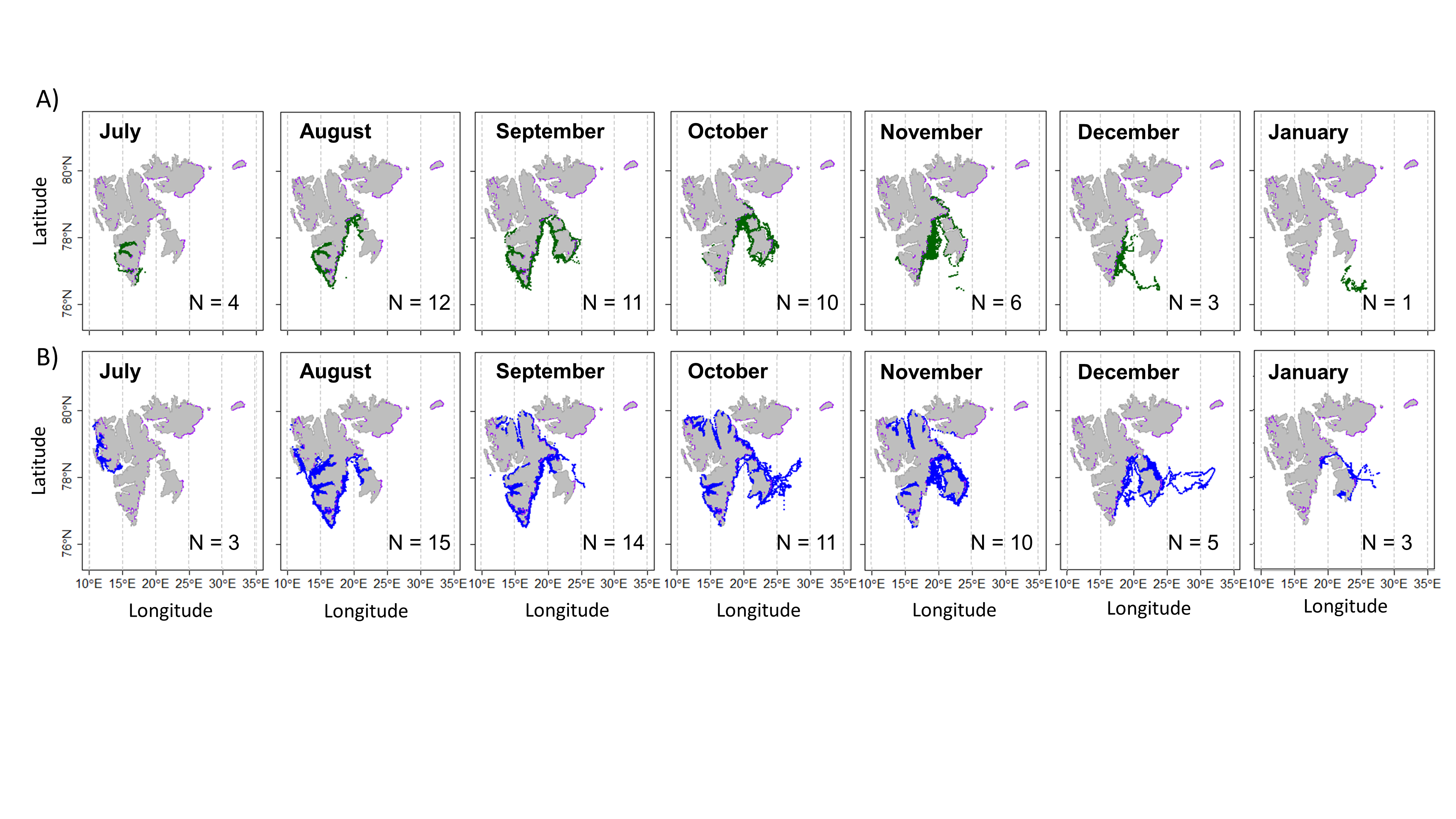

Supplement: Supplementary file 2 — Figure S2. Movements of white whales throughout the first (1995–2001) and the second (2013–2016) tracking periods. Hourly locations of the 34 male white whales tracked in Svalbard, Norway, after the interpolation of the filtered and on-land corrected tracks per month. A) represents the movement of animals throughout the first tracking period (1995–2001) and B) represents the movement of animals throughout the second tracking period (2013–2016). N corresponds to the number of individuals reporting data per month. Purple lines correspond to the tidal glacier front data corresponding to each of the two periods. (TIF 2847 kb) [file 40462_2018_139_MOESM2_ESM.tif]

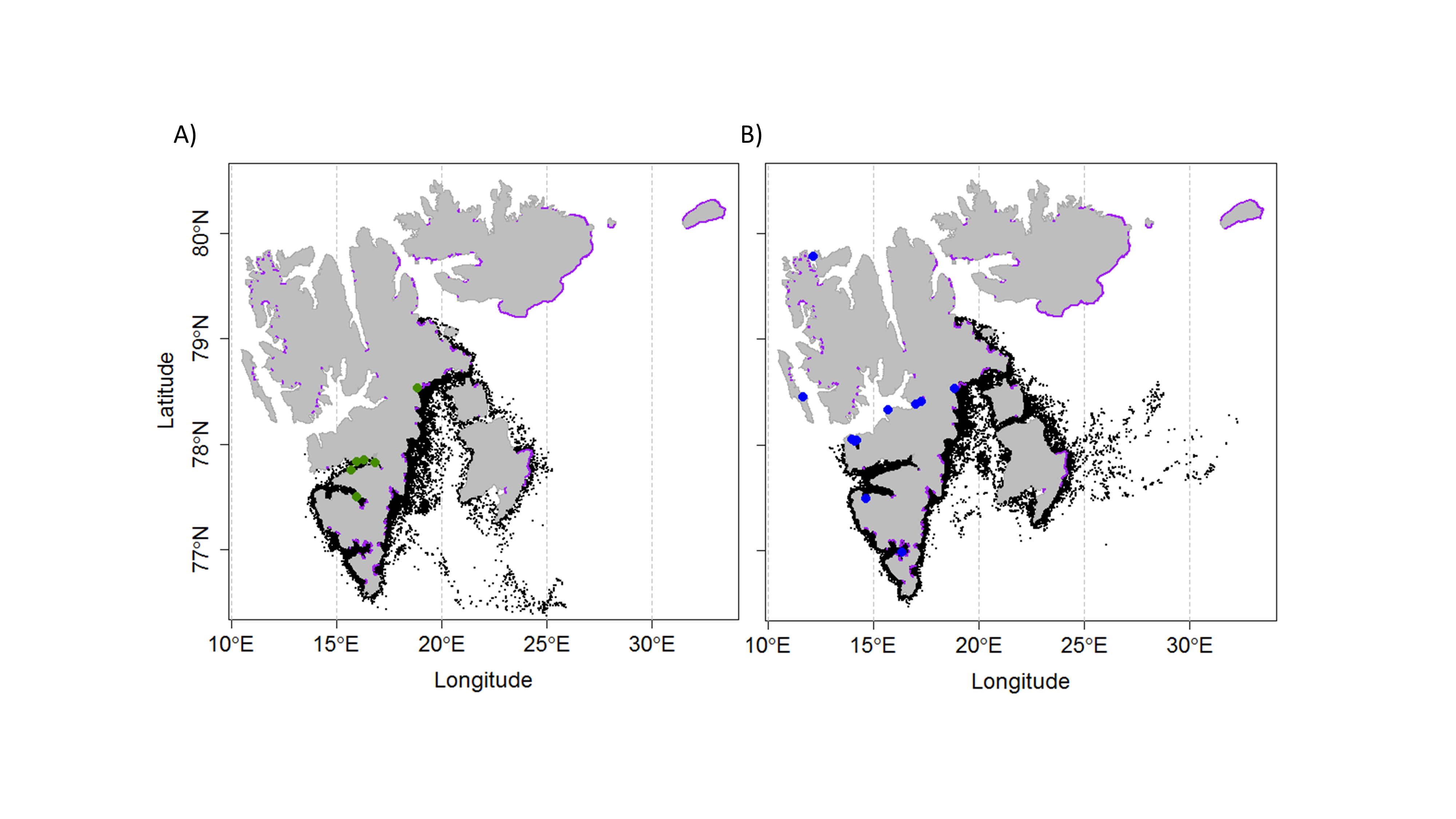

Supplement: Supplementary file 3 — Figure S3. Filtered and corrected tracks for white whales satellite tagged in Svalbard, Norway, during two time periods (excluding the non-common areas between the two periods). Tracks of 34 male white whales equipped with biotelemetry devices in Svalbard, Norway after filtration and correction of on-land positions during A) the period 1995–2001 and B) the period 2013–2016. The blue and green dots represent the deployment points for these two periods. Purple lines correspond to the tidal glacier front data corresponding to each of the periods. (TIF 1923 kb) [file 40462_2018_139_MOESM3_ESM.tif]

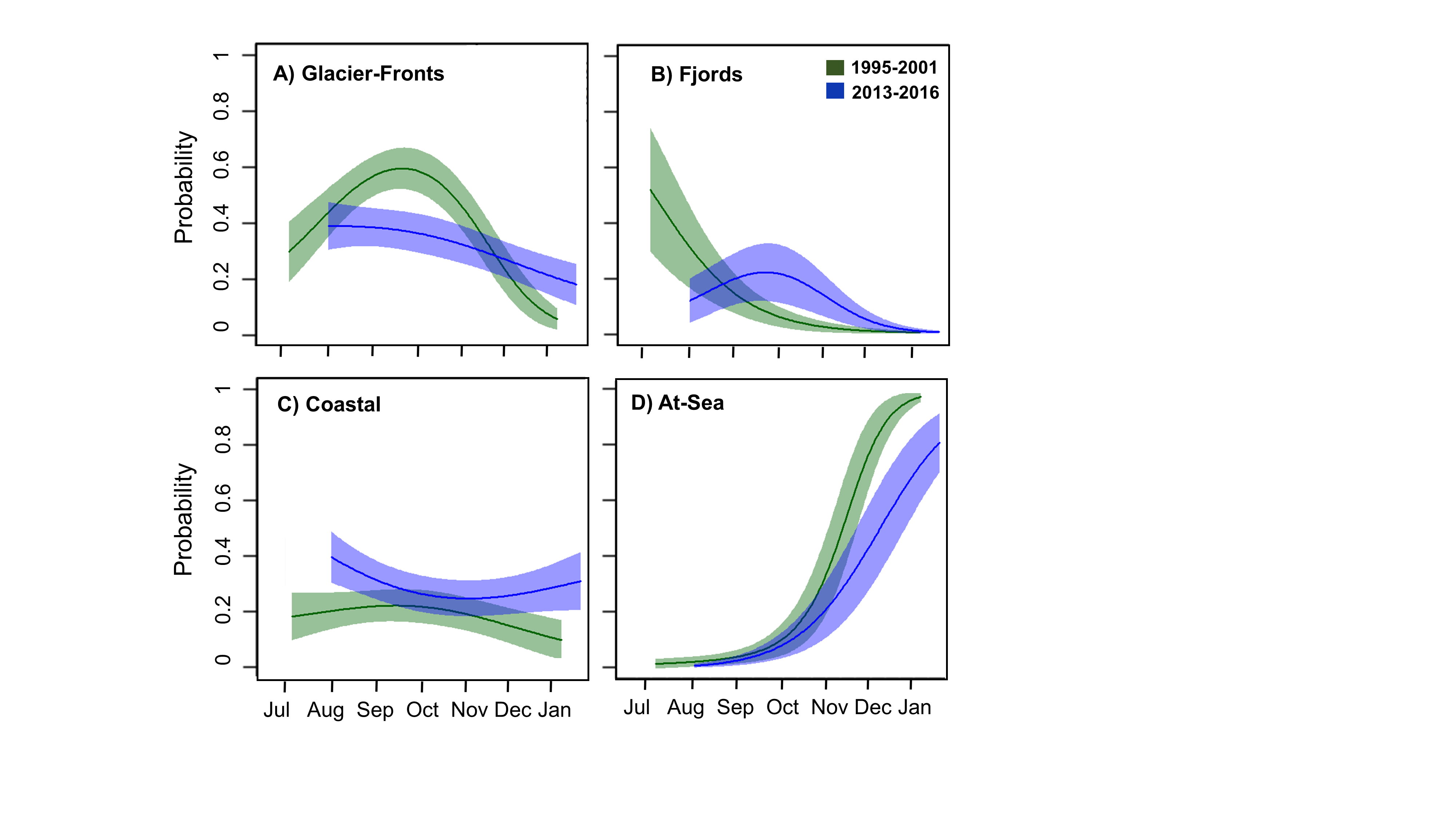

Supplement: Supplementary file 4 — Figure S4. Probability of white whales being in each of four habitat classes by day of year, excluding the non-common areas between the two periods. Results of generalized additive mixed effects models showing the probability of white whales being in Glacier-Front (A), Fjord (B), Coastal (C) or At-Sea (D) habitat classes in Svalbard, Norway, according to day of the year during the first (1995–2001 -green) and the second (2013–2016 - blue) time periods. Values shown are mean ± 95% CI. (TIF 1035 kb) [file 40462_2018_139_MOESM4_ESM.tif]
